# Ionic Liquid Electrolytes for Li–Air Batteries: Lithium Metal Cycling

**DOI:** 10.3390/ijms15058122

**Published:** 2014-05-08

**Authors:** Lorenzo Grande, Elie Paillard, Guk-Tae Kim, Simone Monaco, Stefano Passerini

**Affiliations:** 1 Institute of Physical Chemistry and Münster Electrochemical Energy Technology (MEET) Battery Research Center, University of Muenster, Corrensstraße 28-30, Muenster 48149, Germany; E-Mails: lorenzo.grande@uni-muenster.de (L.G.); gkim0_01@uni-muenster.de (G.-T.K.); 2 Dipartimento di Chimica Giacomo Ciamician, Alma Mater Studiorum University of Bologna, via Selmi 2, Bologna 40126, Italy; E-Mail: simone.monaco2@unibo.it; 3 Helmholtz Institute Ulm, Karlsruhe Institute of Technology, Albert-Einstein-Allee 11, Ulm 89081, Germany

**Keywords:** lithium air batteries, ionic liquids, lithium metal, electrolytes, Pyr_14_TFSI, efficiency calculation, solid electrolyte interphase (SEI)

## Abstract

In this work, the electrochemical stability and lithium plating/stripping performance of *N*-butyl-*N*-methylpyrrolidinium bis(trifluoromethanesulfonyl)imide (Pyr_14_TFSI) are reported, by investigating the behavior of Li metal electrodes in symmetrical Li/electrolyte/Li cells. Electrochemical impedance spectroscopy measurements and galvanostatic cycling at different temperatures are performed to analyze the influence of temperature on the stabilization of the solid electrolyte interphase (SEI), showing that TFSI-based ionic liquids (ILs) rank among the best candidates for long-lasting Li–air cells.

## 1. Introduction

Electric and hybrid cars have successfully entered the market in the last few years, while renewable energy sources, like wind turbines and solar panels, are being deployed at a fast pace. However, the intermittency of these energy sources hinders the possibility to provide a stabilized current baseline for electric grid operation, and batteries with improved performances are actively investigated to meet this shortcoming, as well as for powering electric vehicles. The secondary Li–air battery, first proposed by Abraham *et al.* [1], is seen as a promising candidate for next generation energy storage systems [2,3], given its high theoretical specific capacity (1168 and 3861 mAh·g^−1^ in the discharged and charged state, respectively). Energy densities as high as 3154 Wh·kg^−1^ can be achieved assuming a practical discharge voltage of 2.7 V [4–7] and considering the bulk of the discharged product formed with the following electrochemical reaction:

(1) $$2\;\text{Li}+{\text{O}}_{2}↔{\text{Li}}_{2}{\text{O}}_{2}$$

**Figure 1.**
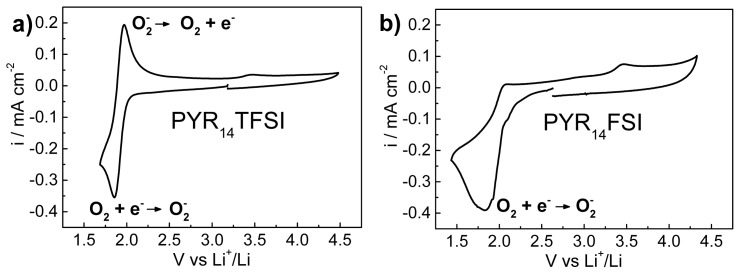
Voltamperogram at 20 mV s^−1^ of (**a**) *N*-butyl-*N*-methylpyrrolidinium bis(trifluoromethanesulfonyl)imide (Pyr_14_TFSI) and (**b**) Pyr_14_FSI; Working electrode: Glassy carbon; Counter electrode: Pt wire; Reference electrode: Ag^+^/Ag.

For practical capacities of around 3 Ah per gram of carbon (a reasonable candidate as a cathode substrate/current collector) [4], this corresponds to a maximum practical specific energy of 2270 Wh·kg^−1^, a value that is above any other existing secondary battery technology. While problems, like O_2_ diffusion [8], pore clogging [9] and low faradaic efficiency, still need to be addressed on the cathode side, a far more important and intrinsic concern is related to the use of volatile solvents as electrolytes in a battery that needs to be open to the atmosphere to harvest and release O_2_. Since lithium metal is present in the system, cell drying poses a risk that should definitely be avoided. Moreover, most reported tests involve either alkyl carbonate- or ether-based electrolytes, which are not stable enough in oxidation [10–13], or solvents, like acetonitrile [12,14] and dimethyl sulfoxide (DMSO) [6], whose stability towards the Li metal electrode is questionable. Ionic liquids (ILs) might bring a solution to this conundrum, thanks to their negligible vapor pressure, high thermal and electrochemical stability, as well as their non-flammability [15]. There is a nascent piece of literature on the subject, which began with a pioneering work on imidazolium-based ionic liquids by Kuboki *et al*. using 1-ethyl-3-methylimidazolium paired with bis(trifluoromethanesulfonyl)imide (TFSI^−^) [16] that was later reprised [17,18], although this class of cations has been shown not to be stable *vs.* lithium metal. Among other ionic liquids, it is worth mentioning the use of diethylmethylmethoxyethylammoniumTFSI, *N*-methoxyethyl-*N*-methylpiperidiniumTFSI [19,20] and *N*-ethyl-*N*-methylpiperidiniumTFSI [19–21]. *N*-butyl-*N*-methylpyrrolidinium (Pyr_14_^+^)-based ILs, such as Pyr_14_TFSI [22] and Pyr_14_FSI [23–25], meet the aforementioned criteria and could be viable candidates for the replacement of conventional electrolytes. Pyr_14_FSI allows high voltage Li–ion cathode use [26], but is not stable *vs.* the very reactive superoxide anion, O_2_^−^, as evidenced by the lack of an O_2_^−^ oxidation peak (Figure 1b), thus ruling out its use in Li–air cells. Pyr_14_TFSI, on the other hand, is more thermally robust and, most importantly, more stable *vs.* O_2_^−^) [27,28], as shown by Figure 1a, which allows Li–O_2_ cell operation [4,8].

In addition to their stability in the presence of O_2_^−^, another issue to be addressed is the viability of Li metal as the negative electrode, its theoretical capacity being more than ten times that of graphite (3861 *vs*. 372 mAh·g^−1^), even though its lower Coulombic efficiency imposes the use of over-stoichiometric Li metal electrodes for secondary battery applications. So far, only polyethylene oxide (PEO)-based solid polymer electrolytes (SPEs) have allowed Li metal to be commercially used in secondary batteries and electric cars, but ionic liquids [25,29] and ionic liquid-containing SPEs [30–32] have already proven successful in several laboratories. For liquid electrolytes, the best efficiencies and, in general, the best performance have been obtained at low to medium temperatures (20 and 40 °C) using a Pyr_14_FSI-based electrolyte [25,29]. In practice, however, this electrolyte poses serious thermal stability issues when the operating temperature is >80 °C [33]. Combinations of TFSI and some quaternary ammonium cations are also valid candidates for Li electrode operation given their high cathodic stability [28,34]. Howlett *et al.* [35] proposed the use of Pyr_14_TFSI-based electrolytes in combination with a Li metal electrode in 2004 and reported high efficiencies for the Li plating/stripping process. The average faradaic efficiency determination usually consists of plating a given amount of Li onto a substrate, such as Cu [35] or Ni [25], equal to a capacity, *Q*_p_, and then cycling a fraction of it (defined as *Q*_c_), until a certain voltage is reached after *n* cycles, which indicates full lithium consumption. Then, either Equation (2) [35] or Equation (3) [25] are used to calculate the average efficiency for one plating/stripping cycle:

(2) $${Eff}_{1}=1-\frac{Q_{\text{p}}}{nQ_{\text{c}}+Q_{\text{p}}}$$

(3) $${Eff}_{2}=1-\frac{Q_{\text{p}}}{nQ_{\text{c}}}$$

While it is obvious that Equation (2) gives higher efficiency results (as Equation (3) considers the first plating to be 100% efficient), the two formulae become equivalent for high efficiencies. Equation (3) was used to report Li plating/stripping efficiencies in Pyr_14_TFSI-based electrolyte on both Pt and Cu [35], although only the use of platinum led to a good cyclability, and less than 50 cycles were reported using copper, a fact that was attributed to the poor initial plating. Most importantly, the fact that lithium alloys with platinum were overlooked makes the results difficult to compare. As a matter of fact, plating large capacities of Li leads to significant volume changes in the electrode and, thus, to the continuous cracking of the solid electrolyte interphase (SEI) [36]. If the consequent repair of the SEI is not effective, dendrite formation and, ultimately, internal short circuits will develop. Aside from the absolute values of *Q*_p_ and *Q*_c_, the *Q*_p_/*Q*_c_ ratio is also of paramount importance to correctly interpret the results obtained. For example, if a 330 μm-thick Li foil (corresponding to *Q*_p_ ≈ 68 mAh·cm^−2^) is used to run plating/stripping tests in symmetrical Li/electrolyte/Li cells and only a small fraction of it (*Q*_c_ = 0.025 mAh·cm^−2^) is actually cycled [37], the high *Q*_p_/*Q*_c_ ratio of ≈2700 will yield a mere 45% efficiency, even after 5000 cycles (using Equation (2)), therefore making plating/stripping tests particularly lengthy. A 99% efficiency evaluation can be reached within 1000 cycles only if the *Q*_p_/*Q*_c_ ratio is as low as ten [25,29]. In our case, a value of *Q*_p_/*Q*_c_ = 100 was chosen.

In general, the low temperature performance of liquid electrolytes based on Pyr_14_TFSI is inferior to that of the Pyr_14_FSI analogues, due to its higher viscosity and lower conductivity. Nevertheless, interesting results can be obtained with Li metal electrodes at 20 and 40 °C when Pyr_14_TFSI is incorporated into SPEs [30,31]. Moreover, the higher thermal stability of Pyr_14_TFSI allows its usage at higher temperatures, which can be of interest in a system with rather slow kinetics and slow mass transport of the active species [4]. Keeping electric vehicles in mind, a higher operating temperature would also lower the energy consumption associated with the battery cooling, as heat transfer is more difficult when the difference in temperature with the exterior is small. Pyr_14_TFSI is thus, a priori, a good candidate for use in combination with Li metal electrodes, but tests performed in ionic liquid based-electrolytes have not provided enough evidence for fully assessing the efficiency of the Li plating/stripping process. So far, a mere 70% efficiency has been reported for a simple potentiodynamic plating/stripping on Ni electrodes [38]. Attempts to measure the efficiency with the abovementioned method with Pyr_14_TFSI-based electrolytes have failed in our laboratories [39], due to the difficulty of obtaining reproducible homogeneous Li deposits on Ni without the use of lower stability additives. Herein, we report Li metal cycling performance in a Pyr_14_TFSI-based electrolyte at different temperatures. Symmetrical Li/electrolyte/Li cells were assembled using a 50 μm-thick Li foil, which leads to quite large Li reservoirs (*Q*_p_ ≈ 10 mAh·cm^−2^). As calculated above, this requires long-term cycling (if *Q*_p_/*Q*_c_ = 100) for efficiency determination.

Another aspect considered is the solid electrolyte interphase (SEI) [36], the protective film that is always present on the lithium surface, which should prevent its extensive oxidation. Its composition varies and depends on the environment seen by the electrode. As lithium metal is highly reactive, a “native” SEI is created during lithium extrusion, *i.e.*, as soon as the fresh surface gets in contact with the dry atmosphere in the production step. This interphase further evolves once it gets in contact with most electrolytes [40–43]. As it is known that TFSI-based ILs participate in the SEI formation [35,44–46], this evolution was investigated at different temperatures, together with its influence on the cycling performance, either by storing the cells under open circuit voltage (OCV) conditions for one week or by making use of separators with differing characteristics. The suitability of the method for efficiency determination using Equation (3), when large Li reservoirs are used, is also discussed.

## 2. Results and Discussion

### 2.1. Effect of Temperature on the Evolution of SEI on Lithium Metal in Contact with Pyr_14_TFSI-Based Electrolyte

**Figure 2.**
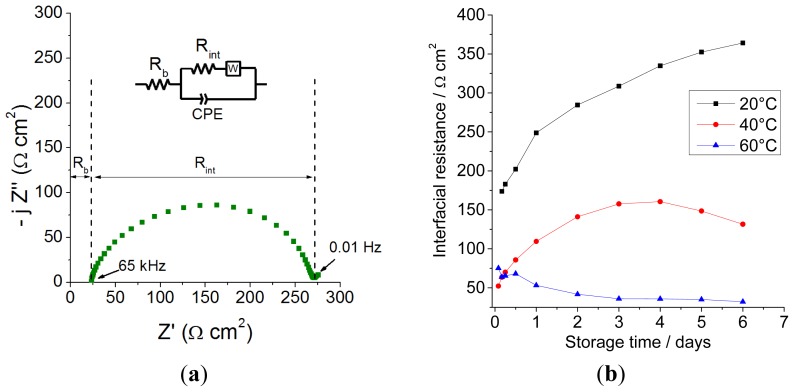
(**a**) Sample Nyquist plot of a Li/0.1 LiTFSI-0.9 Pyr_14_TFSI/Li cell at 20 °C; (**b**) evolution of the interfacial resistance of the cells at 20, 40 and 60 °C.

A sample impedance spectrum for symmetrical Li/0.9 Pyr_14_TFSI-0.1 LiTFSI/Li cells is represented in Figure 2a, together with the associated equivalent circuit. Since it is very difficult to split the contribution of the charge transfer resistance from that of the SEI, the two Randles cells have been merged into one that accounts for the overall interfacial resistance (*R*_int_).

A set of Li/electrolyte/Li cells were thermally stabilized for one week (168 h) under OCV conditions and their evolution of *R*_int_ compared (Figure 2b). A different trend can be seen for each temperature during the same timescale: at 20 °C, *R*_int_ increases constantly, indicating a moderate interfacial reactivity; at 40 °C, the lower initial resistance (as a consequence of a higher temperature) reaches a maximum after 3–4 days (probably due to faster kinetics) and then decreases; finally, at 60 °C, a steady drop is observed for the whole investigated timespan, indicating that the maximum resistance is reached even before the first measurement is performed (*i.e.*, after 2 h). Under these conditions, a stable SEI forms within only three days. This behavior can be interpreted as the result of a progressive accumulation of decomposition products at the Li/electrolyte interface, which goes on until the entire lithium surface is completely covered with a mixed inorganic/organic SEI; our tests show that the higher the temperature, the faster this step will reach completion. The decrease, on the other hand, reflects either the dissolution of SEI components or the swelling of the organic external SEI layer, which leads to its gelation/plasticization [47]. This assumption is realistic for an SEI, where a pure Li^+^ ion conductive (thus, ceramic-like) layer is present close to the Li metal surface, while further away (where the reducing conditions are less stringent), a more organic/polymeric layer forms [44], as postulated by others for ethylene carbonate (EC):dimethyl carbonate (DMC)-based electrolytes [48]. Moreover, the presence of this additional film helps explain the diffusion phenomena occurring within the SEI, for which a pure Li^+^ conducting layer cannot be accounted.

### 2.2. Effect of the Temperature on the Voltage Profiles

**Figure 3.**
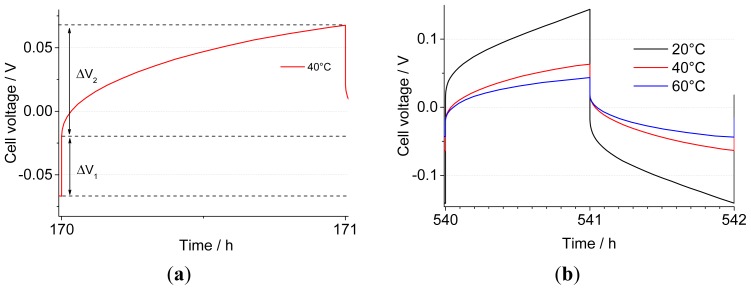
(**a**) Illustration of the quantities, ΔV_1_ and ΔV_2_, in a sample voltage profile; (**b**) comparison of the voltage profiles for cells thermally conditioned for 168 h (one week) at different temperatures, as indicated on the graph.

The cells subjected to a one-week stabilization, as well as the freshly assembled ones, were galvanostatically cycled at a current density of 0.1 mA·cm^−2^, which was reversed every hour. The resulting voltage *vs*. time curve can be analyzed by splitting the component related to the ohmic drop (Δ*V*_1_) from the one linked to the establishment of a diffusion gradient (Δ*V*_2_), as indicated for a sample half-cycle in Figure 3a. The potential increases with the square root of time as expected at constant current according to the Sand equation [49]; this reflects the concentration polarization of the lithium salt arising from a lithium transport number lower than unity. A full depletion of the lithium salt was not observed in this case. From Figure 3b, one can see that the decrease in cell voltage with temperature is in good agreement with an increase of the Li^+^ diffusion coefficient and that no full Li depletion is reached at the plated electrode. To follow the evolution of the system during cycling, in addition to the equivalent series resistance (ESR = Δ*V*_1_/2*i*), it is possible to define a diffusion-related resistance, corresponding to the additional polarization at the end of each step (*R*_pol_ = Δ*V*_2_/2*i*), to account for the additional Li mass transport resistance after one hour. The same quantities can be obtained during the reverse plating.

### 2.3. Effect of Thermal Conditioning on the Cycling Performance

**Figure 4.**
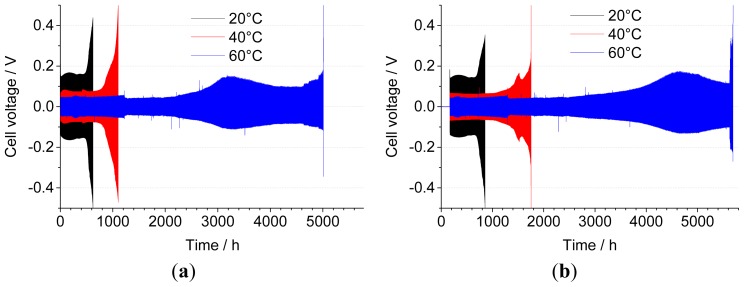
Galvanostatic cycling of Li/0.1 LiTFSI-0.9 Pyr_14_TFSI/Li cells at 0.1 mA·cm^−2^ at 20, 40 and 60 °C. (**a**) Without thermal conditioning; (**b**) including one week of thermal conditioning at open circuit voltage and cycling temperature. Separator: Whatman GF-F.

Figure 4a shows the evolution of the maximum cell voltage recorded during cycling of a set of “fresh” Li/0.9 Pyr_14_TFSI-0.1 LiTFSI/Li cells assembled using a Whatman GF-F separator at different temperatures. As can be seen in Table 1, there is a strong correlation between temperature and the number of cycles/efficiency, with 308 cycles (and 67.5% efficiency) reached before cut-off at 20 °C and 2503 cycles (and 96% efficiency) at 60 °C. According to the assumption behind Equations (2) and (3), one (or both) Li electrode is fully consumed at the end of the cycling test, due to parasitic reactions occurring at the interface with the electrolyte. If this were the case, one would expect an interfacial reactivity (and, thus, cycle inefficiency) scaling up with temperature. On the contrary, visual observation of the two electrodes at the end of cycling confirms that the Li foil is not fully consumed, and the backside of the electrode still has a shiny and metallic appearance. Therefore, the efficiency values calculated here represent an underestimation of the real capabilities of the system: the 0.5 V cut-off voltage is only reached because of the increasing cell resistance upon cycling rather than due to full Li depletion. The progressive increase of the end-of-cycle voltage, at 20 and 40 °C, is in good agreement with this explanation, while at 60 °C, the final increase occurs rather abruptly.

If we compare the previous results with those obtained with thermally conditioned cells, shown in Figure 4b, it is clear that the trend with temperature is the same and that the thermal conditioning has a positive effect in terms of profile regularity (*i.e.*, less fluctuations of cell overvoltage over time) and cycling stability at any temperature. While this beneficial influence is more marked at 40 °C than 20 °C, thanks to the faster interface stabilization, the same cannot be said for cells running at 60 °C, where one week of stabilization only brings in 400 more cycles, corresponding only to a small increase in efficiency (+0.5%). The reason for this lies in the fact that “fresh” cells are also thermally equilibrated for 12 h prior to cycling. At 60 °C, this is long enough to create a stable SEI (as already shown in Figure 2a); thus, little improvement can be achieved with longer storage times, unlike at lower temperatures.

### 2.4. Analysis of the Cell Resistances

**Figure 5.**
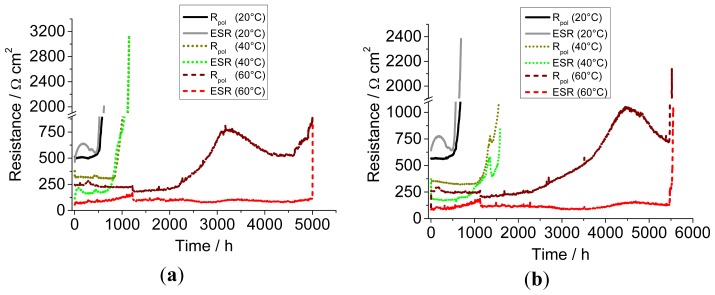
Evolution of *R*_pol_ and equivalent series resistance (ESR) with cycling time for the set of (**a**) fresh cells and (**b**) thermally conditioned cells.

**Figure 6.**
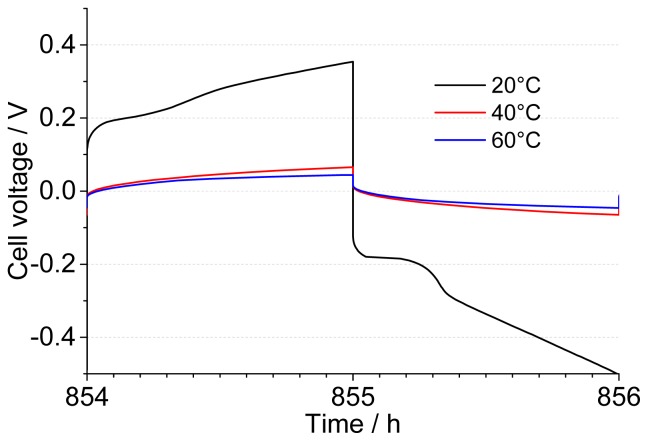
Voltage profile of Li/0.9 Pyr_14_TFSI-0.1 LiTFSI/Li fresh cells subjected to galvanostatic cycling at temperatures as indicated on the graph.

For a better analysis of the cell voltage, the evolution of ESR and *R*_pol_ with cycling for the fresh and conditioned cells has been plotted in Figure 5a,b, respectively. As mentioned earlier, the first term is related to the ohmic drop, while the second arises as a consequence of diffusion-related processes. One can notice that at 20 and 40 °C, the voltage variations, prior to the final increase, are mostly linked to the evolution of ESR, while *R*_pol_ stays rather constant. At 60 °C, after several hundreds of cycles, a peak in *R*_pol_ is observed, followed by a steady decrease and then a final increase, while at lower temperatures, the upward trend in *R*_pol_ is uninterrupted throughout cycling. In the case of the non-stabilized cell at 60 °C, a constant *R*_pol_ value around 500 Ω cm^2^ (Figure 5a) is maintained for *ca*. 250 cycles after 4000 h. In the hypothesis of an increase of mass transport resistance within the bulk electrolyte, we would not expect such a tendency, especially if we consider that, according to Figure 5a,b, ESR does not show a strong correlation with variations in *R*_pol_. The observed trend is, on the other hand, coherent with a mass transport limitation determined by the growth of an outer polymeric SEI or, alternatively, by a mechanical rupture of the outer SEI and its subsequent self-healing. In the latter event, the exposure of a fresh Li/electrolyte interface would trigger the re-establishment of a uniform, equipotential surface [50]. During this time, the fractures would act as preferential channels for the transport of Li^+^ ions, thereby countering the polarization induced by the SEI. This is not observed at 20 and 40 °C, because the voltage cut-off is reached before any decrease in *R*_pol_ can be achieved. A careful analysis of the voltage profiles during the final cycles gives a more insightful picture (Figure 6).

During Li plating, the polarization appears like a single, diffusion-controlled electrochemical process, as shown in Figure 3, without Li depletion. However, as the voltage profile evolves into more complex shapes with time, the cell at 20 °C shows a further potential rise after an initial steady state (Figure 6). Finally, after several hundred cycles, the large potential deviations give rise to a Sand behavior, but not to a total salt depletion. At 40 and 60 °C, such a behavior is only seen several hundred cycles later, indicative of the increase in the salt diffusion coefficient with temperature. The two diffusion-controlled processes appear to be partially overlapped. According to our interpretation, once a concentration gradient is established in the electrochemical cell, the variation in Li^+^ concentration within the SEI remains negligible, as long as it is “thin” enough, *i.e.*, it behaves in an ideal way. When the SEI thickness (and, therefore, its resistance) increases over a critical value, a concentration gradient can establish within this interphase and show up in the voltage *vs*. time chart (as reported in Figure 6). It should be pointed out that the shape of the voltage profile can also be linked to parasitic reactions, such as Pyr_14_TFSI decomposition at a low potential, but the pieces of evidence gathered so far, such as the decrease of *R*_int_ at 40 and 60 °C over storage time and the lowering of *R*_pol_ over cycling, clearly show that the electrolyte is stable enough *vs.* lithium and does not incur massive decomposition. In either case, once a given value of the cell voltage (different for each temperature) is breached, an increased clogging of the electrodes takes place, slowing down Li^+^ transport and leading to the end of cycling, as evidenced by the fast final increase of ESR and *R*_pol_. It must be stressed once again that this does not correspond to full Li consumption, but is related to the reactivity of the electrolyte at the Li interface. As stated previously, the native SEI formed in contact with the atmosphere is mainly composed of inorganic compounds (Li_2_CO_3_, Li_2_O, Li_3_N) that form microcrystalline or microphase domains [51]. This surface heterogeneity induces an inhomogeneous current distribution, due to the different size of the domains, different conductivity and faster Li^+^ ion transport at grain boundaries. Electrolyte components can infiltrate along the grain boundaries and reach the close vicinity of the Li metal, especially through domains where the SEI is less resistive and less protected. With time, the accumulation of these products blocks all the diffusion channels and leads to the final increase in the plating overvoltage.

### 2.5. Effect of the Separator on the SEI Evolution

Damage to the lithium surface can also arise after contact with the fibers that make up the separator. These can break the SEI surface similarly to what is believed to occur for polymer electrolytes [51]. To confirm this, a glass fiber separator (Whatman GF-C, 260 μm thickness) with slightly different properties compared to the Whatman GF-F (420 μm thickness) was tested.

**Figure 7.**
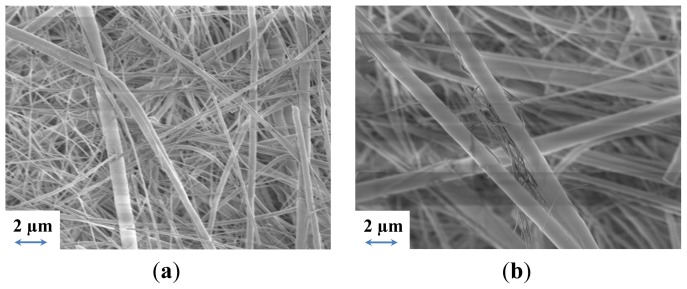
SEM micrographs of (**a**) Whatman GF-F and (**b**) GF-C separators.

**Figure 8.**
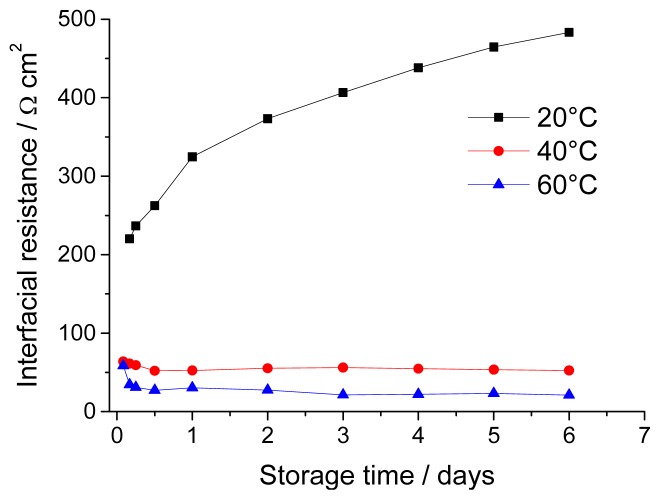
Evolution of the interfacial resistance of the cells at 20, 40 and 60 °C. Separator: Whatman GF-C.

GF-C allows a faster Herzberg liquid flow, thanks to its larger pore size (1.2 *vs*. 0.7 μm for GF-F). In addition, its lower thickness leads to an overall lower resistance and improved Li^+^ mass transport. The structure of the two separators are similar, as both are non-woven mats (as seen in the SEM images in Figure 7), but the GF-C separator is expected to have a lower fiber surface in direct contact with the Li surface, in accordance with the larger pore size and the larger size of its constitutive fibers. The minor impact on the “native” SEI could therefore lead to limited reactivity [51]. The trend of the interfacial resistance change over time for cells using a GF-C separator was monitored for a week, as shown in Figure 8.

For the cell stored at 20 °C, the SEI resistance grows gradually like with GF-F separators, while a decrease and fast stabilization takes place at 60 °C and, unlike the previous tests, at 40 °C, as well. This observation goes hand in hand with the interpretation that a lower surface area of the separator in contact with the Li electrode causes less damage on the SEI and the deposition of a smaller number of degradation products onto the affected areas. More importantly, the restricted generation of partially reduced soluble products within the electrolyte also leads to the formation of an overall thinner SEI. In the GF-C cell stored at 40 °C, a local and fast SEI repair occurs, as the few cracked parts are very reactive. No difference in the use of a different separator is seen at 60 °C, since the reaction kinetics is fast enough to seal all cracks and limit the formation of soluble products. Finally, at 20 °C, the kinetics is still sluggish, even if a thinner, more porous separator is used, and the few cracks formed can still trigger the generation of lower solubility products and their accumulation.

### 2.6. Effect of the Separator on the Cycling Performance

**Figure 9.**
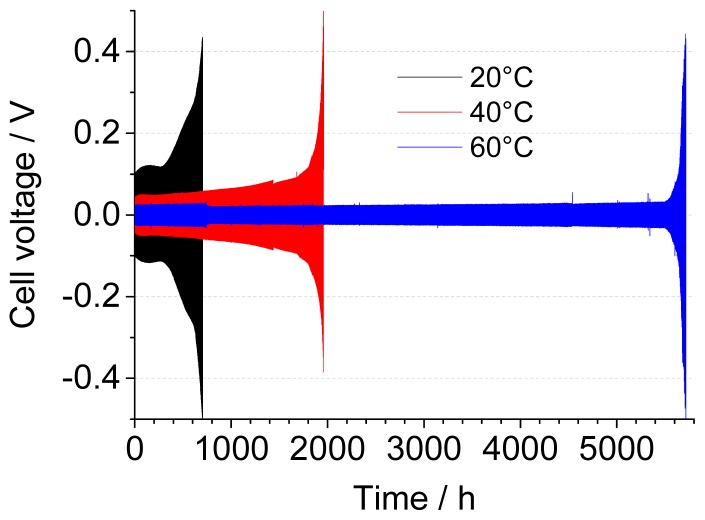
Galvanostatic cycling of “fresh” Li/0.1 LiTFSI-0.9-Pyr_14_TFSI/Li cells at 0.1 mA·cm^−2^ at 20, 40 and 60 °C. Separator: Whatman GF-C.

In order to investigate if the faster stabilization of the SEI has any effect on the cycling performance, “fresh” Li/Li symmetrical cells were assembled at different temperatures and cycled, in the same condition as before, and the voltage profile curves are shown in Figure 9.

An improvement in terms of cycles is observed in all three tests, similarly to the case of the conditioned cells; Table 2 lists the number of cycles and efficiencies obtained by each cell, as well as the percentage increase with respect to the corresponding GF-F fresh cells. The improvement of performances, in terms of cell voltage, profile regularity and cycles reached, justify our assumptions on the overall higher electrolyte stability due to less SEI clogging, especially the marked enhancement recorded at 40 °C. This result has also been confirmed with separators having smoother surfaces, such as Celgard 2500, which lead to lower SEI resistance [52]. Finally, it must be noted that similarly to the case of GF-F, the electrode clogging, rather than full Li consumption, is responsible for the end of cycling, also with GF-C separators.

## 3. Experimental Section

### 3.1. Materials Used

Lithium metal foil (50 μm, 99.999%, Rockwood Lithium GmbH, Frankfurt am Main, Germany) was stored in a dry room (Relative Humidity <0.1% at 20 °C) together with all the other chemicals mentioned in this paper. *N*-butyl-*N*-methylpyrrolidinium bis(trifluoromethanesulfonyl)imide (Pyr_14_TFSI) was synthesized according to a well-established procedure [46] and dried at 90 °C for at least 24 h using a turbomolecular pump (vacuum <10^−7^ mbar). This procedure leads to ILs exhibiting water content below 2 ppm, measured via coulometric Karl Fischer titration (C30, Mettler Toledo, Schwerzenbach, Switzerland). Lithium bis(trifluoromethanesulfonyl)imide (LiTFSI) (99.9%, battery grade, 3M, St. Paul, MN, USA) was dried at 120 °C under vacuum for at least 24 h before use. Pyr_14_TFSI and LiTFSI were mixed in a 9:1 molar ratio and further dried under turbomolecular vacuum at 70 °C for 24 h. Glass fiber separators (Whatman GF-F and GF-C, GE Healthcare UK Ltd., Little Chalfont, UK) were dried at 120 °C for at least 24 h prior to usage.

### 3.2. Cyclic Voltamperometry

The oxygen redox reaction was investigated by cyclic voltammetry (CV) using a glassy carbon electrode (3 mm diameter, Tokai Carbon Co., Ltd., Tokyo, Japan), in a 5-mL cell, which was thermostated at 30 °C by a K40 thermocryostat (Haake Technik GmbH, Vreden, Germany). The reference electrode was a silver wire in 6 × 10^−2^ M AgTFSI (97%, Sigma-Aldrich, St. Louis, MO, USA) -PYR_14_TFSI. The reference electrode potential was checked *vs*. lithium, and the working potentials are reported *vs*. the Li^+^/Li couple. A Pt wire was used as a counter electrode. The voltammetric scans were corrected for the ohmic drop evaluated by impedance spectroscopy in the 10 kHz–1 Hz range. These tests were performed using a VSP multichannel potentiostat/galvanostat/electrochemical impedance analyser, (PerkinElmer, Waltham, MA, USA).

### 3.3. Cell Preparation

All cell assembly procedures were performed inside the dry room. Pouch bags were manufactured using two 100 μm “coffee-bag” sheets and Ni current collectors (thickness: 20 μm). Lithium foil strips were then folded around the current collectors to ensure electrical contact and separated by a glass fiber separator impregnated with the electrolyte. The cells thus fabricated had an active area of *ca*. 1 cm^2^.

### 3.4. SEI Resistance Evolution

Some of the pouch cells were subjected to a one-week thermal conditioning, by storing them in a MK53 climatic chamber Δ*T* = ±0.1 °C (Binder GmbH, Tuttlingen, Germany) at three different temperatures (20, 40 and 60 °C) and electrochemical impedance measurements were acquired daily to track the changes in interfacial resistance over time. For this purpose, the range spanning from 65 kHz to 10 MHz was investigated, using a Solartron 1287 potentiostat/galvanostat coupled to a Solartron 1260 frequency response analyzer (Solartron Analytical, Farnborough, UK).

### 3.5. Galvanostatic Cycling

All pouch cells were galvanostatically cycled using a battery cycler (S4000, Maccor Inc., Tulsa, OK, USA) by applying a 0.1 mA·cm^−2^ current density that was reversed every 60 min. Cut-off voltages of 0.5 and −0.5 V were used. Thermal equilibration was ensured by inserting a 12-h rest step before performing each test.

### 3.6. Scanning Electron Microscopy

The morphology of the glass fiber separators was analyzed via high-resolution scanning electron microscopy (SEM, AURIGA^®^ microscope, Zeiss, Oberkochen, Germany). The sample surfaces were made conductive by using a turbo-pumped gold sputter/coater (Quorum Q150T, Quorum Technologies Ltd., Co., East Grinstead, UK). The current applied was 45 mA for 30 s.

## 4. Conclusions

In this work, we showed that it is possible to achieve cycling efficiencies as high as 96.5% through plating/stripping tests of lithium metal electrodes in a Pyr_14_TFSI-based electrolyte. Moreover, the calculated average Coulombic efficiency represents an underestimation of the real value, as the end of cycling is neither triggered by full lithium consumption nor by parasitic reactions, but rather by the clogging of all Li^+^ ion diffusion channels in the solid electrolyte interphase (SEI). This phenomenon is slowed down by the formation of an outer polymeric SEI that promotes the stabilization of the Li/electrolyte interface and leads to a delayed clogging of the electrode, as less degradation products are generated during cycling. The stabilization of SEI becomes more effective at higher temperatures, an effect that is enhanced by the faster Li^+^ ion transport within the electrolyte. The study of two different glass fiber separators with the same chemical composition also showed that the porosity and morphology of the separator can influence the “native” SEI’s open circuit evolution and the electrode clogging upon cycling. These features pave the way to the usage of ionic liquid electrolytes in next-generation electrochemical energy storage technologies, such as Lithium–air batteries. The long-term cyclability that IL-based electrolytes ensure, together with the peculiar physico-chemical properties, like negligible volatility and wide electrochemical stability, are key enabling factors to the deployment of such novel electrochemical power sources into the market.

## Figures and Tables

**Table 1.** Number of cycles before cut-off voltage is reached and corresponding efficiencies (Eff_2_), according to Equation (3) (*Q*_p_ = 10 mAh cm^−2^, *Q*_c_ = 0.1 mAh cm^−2^), for two sets of Li/0.9 Pyr_14_TFSI-0.1 LiTFSI/Li cells.

	20 °C		40 °C		60 °C	
			
	Cycles	Eff_2_	Cycles	Eff_2_	Cycles	Eff_2_
GF-F Fresh (12 h)	308	67.5%	549	81.8%	2503	96.0%
GF-F Conditioned (168 h)	344 (+11.8%)	70.9% (+3.4%)	793 (+44%)	87.4% (+5.6%)	2897 (+15%)	96.5% (+0.5%)

**Table 2.** Number of cycles before the cut-off voltage is reached and corresponding efficiencies, according to Equation (3) (*Q*_p_ = 10 mAh·cm^−2^, *Q*_c_ = 0.1 mAh·cm^−2^), for a set of Li/0.9 Pyr_14_TFSI-0.1 LiTFSI/Li cell including a GF-C separator.

	20 °C		40 °C		60 °C	
			
	Cycles	Eff_2_	Cycles	Eff_2_	Cycles	Eff_2_
GF-C Fresh (12 h)	351 (+14%)	71.5% (+4%)	980 (+78%)	89.8% (+8%)	2858 (+14%)	96.5% (+0.5%)
